# Indirect Interspecies Regulation: Transcriptional and Physiological Responses of a Cyanobacterium to Heterotrophic Partnership

**DOI:** 10.1128/mSystems.00181-16

**Published:** 2017-03-07

**Authors:** Hans C. Bernstein, Ryan S. McClure, Vera Thiel, Natalie C. Sadler, Young-Mo Kim, William B. Chrisler, Eric A. Hill, Donald A. Bryant, Margaret F. Romine, Janet K. Jansson, Jim K. Fredrickson, Alexander S. Beliaev

**Affiliations:** a Biological Sciences Division, Pacific Northwest National Laboratory, Richland, Washington, USA; b Environmental Molecular Sciences Laboratory, Pacific Northwest National Laboratory, Richland, Washington, USA; c Department of Biochemistry and Molecular Biology, The Pennsylvania State University, University Park, Pennsylvania, USA; d Department of Chemistry and Biochemistry, Montana State University, Bozeman, Montana, USA; e The Gene and Linda Voiland School of Chemical Engineering and Bioengineering, Washington State University, Pullman, Washington, USA; Northwestern University Feinberg School of Medicine

**Keywords:** consortia, cyanobacteria, heterotroph, microbial interactions, transcriptome

## Abstract

The mechanisms by which microbes interact in communities remain poorly understood. Here, we interrogated specific interactions between photoautotrophic and heterotrophic members of a model consortium to infer mechanisms that mediate metabolic coupling and acclimation to partnership. This binary consortium was composed of a cyanobacterium, *Thermosynechococcus elongatus* BP-1, which supported growth of an obligate aerobic heterotroph, *Meiothermus ruber* strain A, by providing organic carbon, O_2_, and reduced nitrogen. Species-resolved transcriptomic analyses were used in combination with growth and photosynthesis kinetics to infer interactions and the environmental context under which they occur. We found that the efficiency of biomass production and resistance to stress induced by high levels of dissolved O_2_ increased, beyond axenic performance, as a result of heterotrophic partnership. Coordinated transcriptional responses transcending both species were observed and used to infer specific interactions resulting from the synthesis and exchange of resources. The cyanobacterium responded to heterotrophic partnership by altering expression of core genes involved with photosynthesis, carbon uptake/fixation, vitamin synthesis, and scavenging of reactive oxygen species (ROS).

**IMPORTANCE** This study elucidates how a cyanobacterial primary producer acclimates to heterotrophic partnership by modulating the expression levels of key metabolic genes. Heterotrophic bacteria can indirectly regulate the physiology of the photoautotrophic primary producers, resulting in physiological changes identified here, such as increased intracellular ROS. Some of the interactions inferred from this model system represent putative principles of metabolic coupling in phototrophic-heterotrophic partnerships.

## Abstract (precis)

This study elucidates how a cyanobacterial primary producer acclimates to heterotrophic partnership by modulating the expression levels of key metabolic genes. Heterotrophic bacteria can indirectly regulate the physiology of the photoautotrophic primary producers, resulting in physiological changes identified here, such as increased intracellular ROS. Some of the interactions inferred from this model system represent putative principles of metabolic coupling in phototrophic-heterotrophic partnerships.

## INTRODUCTION

Interspecies microbial interactions are controlled by the genome-encoded functions belonging to individual organisms and from their responses to environmental cues. Community-level responses are a function of all species, including those in low abundance, and comprehensive analyses require species-level resolution (1). Most microbial communities in nature are structurally and functionally complex, and it is technically challenging to make species-specific observations of behavior. Hence, model microbial consortia, maintained under controlled environments, are attractive for interrogating the principles by which multispecies interactions mediate the exchange of nutrients, vitamins/cofactors, and energy under different growth conditions and environmental constraints (2–6).

Phototroph-heterotroph partnerships are essentially ubiquitous in photic environments and mediate key biogeochemical and ecological processes on a global scale (7). In this study, we employed a bottom-up approach to infer and test specific interactions occurring within a constructed binary consortium containing a unicellular cyanobacterium, *Thermosynechococcus elongatus* BP-1, and an obligate aerobic heterotroph, *Meiothermus ruber* strain A (here *T. elongatus* and *M. ruber*, respectively). *T. elongatus* is a thermophilic, unicellular cyanobacterium previously investigated in numerous ecophysiological and biotechnological studies (8–13). Its genome is well characterized (14) and was first isolated from a hot spring cyanobacterial mat environment near Beppu, Japan (15). *M. ruber* strain A is an aerobic, heterotrophic thermophile isolated from an enrichment culture of *Synechococcus* sp. strain JA-3-3-Ab, which was sampled from a cyanobacterial mat inhabiting the outflow of Octopus Spring in Yellowstone National Park (WY, USA) (16, 17), and shares 98.6% nucleotide identity to the 16S rRNA gene of a closely related strain, *Meiothermus ruber* DSM 1279 (18). At the genome level, both *M. ruber* strains display substantial functional relatedness with regard to carbohydrate and energy metabolism, including genes for glycolysis, tricarboxylic acid cycle, oxidative pentose phosphate, Entner-Doudoroff, and aerobic respiratory electron transfer pathways (16). Similarly to *M. ruber* DSM 1279, strain A lacks an assimilatory nitrate reduction pathway; hence, it depends upon reduced N sources produced by *T. elongatus* when cocultured in minimal medium containing only nitrate. This consortium was constructed specifically to identify interactions underlying acclimation responses to heterotrophic partnership. Simplified consortia, such as the system presented here, are useful tools for studying microbial interactions at the species level. It is much more difficult to interrogate species-resolved responses to partnership in natural systems, in which membership cannot be controlled. Typically, such studies have invoked only bulk measurements of physiological or biochemical activities derived from the entire community or have examined subsets, focusing on only the dominant members (19).

In this study, analyses were performed on both member species by interrogating transcriptional and physiological data associated with a commensal “producer-consumer” interaction (20). These data were collected across tightly controlled steady states maintained via discrete incident irradiance (I_i_) and dissolved O_2_ tension (pO_2_) treatments. The strains were chosen because of an obligate dependency of *M. ruber* on cyanobacterium-derived carbon and nitrogen, when the consortium was grown in autotrophic minimal medium containing nitrate. These are representative thermophiles derived from hot spring cyanobacterial mats that are hallmark habitats for high solar I_i_ and pO_2_ that significantly influence microbial interactions and the ecosystem properties (21–25). We designed controlled cultivation experiments that compared axenic growth of *T. elongatus* to growth in the consortium and concluded that *M. ruber* acted as a commensal partner supported by direct metabolite exchange. We then hypothesized that *T. elongatus* sensed and acclimated to its partner and that this behavior represented indirect interspecies regulation where each species coordinated some transcriptional events in response to the other. We conclude that some of these behaviors that relate to the most foundational functions of life, such as carbon and energy acquisition, may represent generalizable principles for phototroph-heterotroph interactions that can occur in habitats subjected to a dynamic range of light and oxygen tensions.

## RESULTS

### Photoautotrophically supported binary culture.

*T. elongatus* supported heterotrophic growth of *M. ruber* under photoautotrophic growth conditions (i.e., CO_2_/HCO_3_^−^ as the sole carbon sources) in the defined medium containing nitrate with no vitamin amendments (see Fig. S1 in the supplemental material). Stable compositional and metabolic steady states were maintained under turbidostat control. The relative abundances of *T. elongatus* and *M. ruber* remained stable across the treatments and maintained average values of 90.2% ± 2.0% and 9.1% ± 1.5% cell counts, respectively. Because *M. ruber* lacks the ability to assimilate nitrate (16) (Fig. S2), its capacity to maintain a stable population indicates that a consistent flux of both organic carbon and organic/assimilated nitrogen emanates from the cyanobacterium. Relative extracellular metabolite measurements (i.e., peak area of quantifiable metabolites) confirmed that organic carbon and nitrogen were available for heterotrophic growth and present in the form of citric acid and several amino acids, respectively, although compounds in lower abundance were not accounted for (Fig. S3).

10.1128/mSystems.00181-16.2 FIG S1  (A) A representative confocal micrograph (1 of 40) of cells maintained as a stable, photosynthetically supported binary culture of *T. elongatus* (red; autofluorescence) supporting *M. ruber* (green; SYBR gold). Bar, 10 µm. (B) Relative abundances measured in percentage of cells counted via fluorescence-activated cell sorting. Values represent the means from three independent steady states held under different oxygen tensions (pO_2_ = 0, 0.3, and 0.6 atm-O_2_). Error bars represent ±1 standard deviation. Download FIG S1, PDF file, 0.1 MB. Copyright © 2017 Bernstein et al. 2017 Bernstein et al. This content is distributed under the terms of the Creative Commons Attribution 4.0 International license.

10.1128/mSystems.00181-16.3 FIG S2  Batch growth and nitrate assay. Each data point represents the mean from three biological replicates. The optical densities (OD_600_) of *M. ruber* cells are matched to the *M. ruber* nitrate samples. Error bars represent ±1 standard deviation. The abiotic control experiment revealed no change in nitrate concentration, which remained constant at 106 ± 3 mg liter^−1^ over 48 h. Download FIG S2, PDF file, 0.2 MB. Copyright © 2017 Bernstein et al. 2017 Bernstein et al. This content is distributed under the terms of the Creative Commons Attribution 4.0 International license.

10.1128/mSystems.00181-16.4 FIG S3  Extracellular metabolite measurements of axenic *T. elongatus* (Ax) and the binary culture (Bi). Specific turbidostat steady-state conditions are designated with the following abbreviations: HL, high light (1,995 µmol photons m^−2^ s^−1^); ML, medium light (1,190 µmol photons m^−2^ s^−1^); LL, low light (197 µmol photons m^−2^ s^−1^); HO, high O_2_ (pO_2_ = 0.6 atm); MO, medium O_2_ (pO_2_ = 0.3 atm); LO, low O_2_ (pO_2_ = 0.0 atm). Measurements are the means from two biological replicates with standard errors. Download FIG S3, PDF file, 0.3 MB. Copyright © 2017 Bernstein et al. 2017 Bernstein et al. This content is distributed under the terms of the Creative Commons Attribution 4.0 International license.

10.1128/mSystems.00181-16.5 FIG S4  Concordance of replication shown as the ranges between biological duplicate measurements of mRNA abundances (RPKM) values for 2,479 genes under each steady-state condition for which *T. elongatus* gene expression was analyzed. Specific turbidostat steady-state conditions are designated with the following abbreviations: AX, axenic *T. elongatus*; BC, binary culture; HL, high light (1,995 µmol photons m^–2^ s^–1^); ML, medium light (1,190 µmol photons m^–2^ s^–1^); LL, low light (197 µmol photons m^–2^ s^–1^); HO, high O_2_ (pO_2_ = 0.6 atm); MO, medium O_2_ (pO_2_ = 0.3 atm); LO, low O_2_ (pO_2_ = 0.0 atm). Download FIG S4, PDF file, 0.2 MB. Copyright © 2017 Bernstein et al. 2017 Bernstein et al. This content is distributed under the terms of the Creative Commons Attribution 4.0 International license.

10.1128/mSystems.00181-16.6 FIG S5  Concordance of replication shown as the ranges between biological replicate measurements of mRNA abundances (RPKM) values for 4,492 genes under each steady-state condition for which species-resolved gene expression was analyzed; binary cultivation of *T. elongatus* and *M. ruber*. Specific turbidostat steady-state conditions are designated with the following abbreviations: HL, high light (1,995 µmol photons m^–2^ s^–1^); ML, medium light (1,190 µmol photons m^–2^ s^–1^); LL, low light (197 µmol photons m^–2^ s^–1^); HO, high O_2_ (pO_2_ = 0.6 atm); MO, medium O_2_ (pO_2_ = 0.3 atm); LO, low O_2_ (pO_2_ = 0.0 atm). Replication of transcriptomic analyses was performed in duplicate with the exception of condition HL-LO, for which replication was performed in quadruplicate. Download FIG S5, PDF file, 0.1 MB. Copyright © 2017 Bernstein et al. 2017 Bernstein et al. This content is distributed under the terms of the Creative Commons Attribution 4.0 International license.

**FIG 1:**
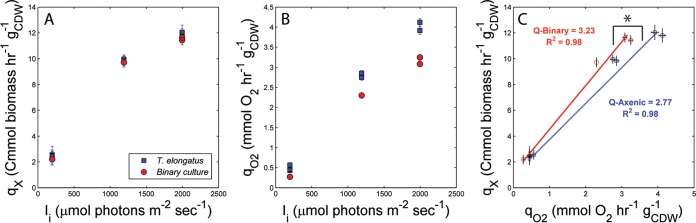
Growth and photosynthesis kinetics of the *T. elongatus* axenic (blue) and binary (red) cultures held under variable irradiance-controlled turbidostat steady states. (A) Specific growth rates on a Cmol basis. (B) Specific rate of net O_2_ production (net photosynthesis). (C) Growth rate plotted against net photosynthesis. The slope of these curves represents the photosynthetic quotients or yields of carbon uptake into biomass per net photosynthetic output. Each data point represents the average of measurements taken every minute over a minimum of three reactor residence times held under each respective steady state. Error bars represent ±1 standard deviation. *, significant differences with >99% certainty as determined by *t* test of unequal sample sizes, assuming equal variance; CDW, cell dry weight.

Turbidostat steady states, controlled by incident irradiance (I_i_), yield the respective maximum specific growth rates (µ) for each condition. Axenic *T. elongatus* and the binary culture µ values increased with I_i_ values that were between 350 and 2,000 µmol photons m^−2^ s^−1^. Cell growth did not saturate or became inhibited within the bounds of experimentally imposed I_i_ treatments. The µ values measured for the binary consortium were nearly identical to those for the *T. elongatus* axenic control under each I_i_ treatment. The cultures (binary and axenic) approached 2.4-h doubling times at the highest I_i_ treatments (2,000 µmol photons m^−2^ s^−1^). As expected, analysis of the specific growth rate, on a carbon-mole basis (Cmmol biomass h^−1^ g^−1^_cell dry weight_), indicated that heterotrophic partnership had a negligible effect on the net conversion of inorganic carbon to biomass compared to the *T. elongatus* axenic control (Fig. 1A). The specific rates of O_2_ production measured in the binary consortium were lower than those in the *T. elongatus* axenic control (Fig. 1B). Hence, the photosynthetic quotients, approximated here as the Cmol fixed into biomass by moles O_2_ produced, were greater in the binary culture than in the *T. elongatus* axenic control (Fig. 1C). The binary culture captured more of its reductant in biomass than did axenic *T. elongatus* as a result of concurrent heterotrophic growth, inferred to be supported by cyanobacterium-derived reduced carbon and nitrogen sources.

### Interaction leads to reduced oxygen sensitivity.

**FIG 2:**
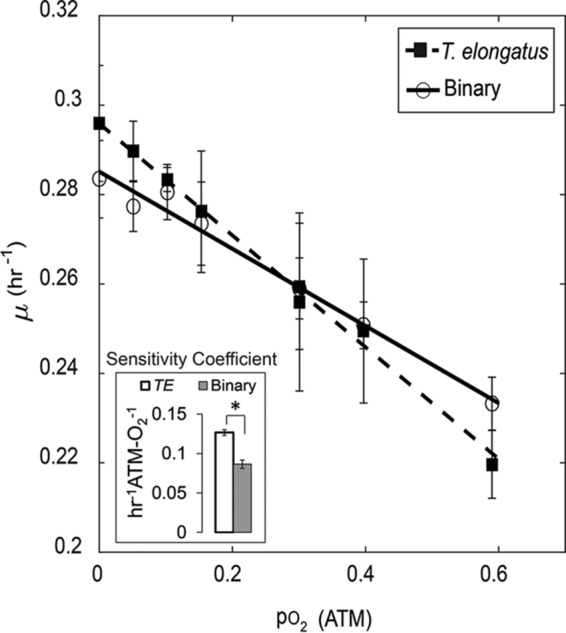
Oxygen inhibition profiles of steady-state specific growth rates controlled under increasing oxygen tensions (pO_2_ of in-gas). The absolute value of the slopes represents the sensitivity coefficients which are inversely proportional to the growth resistance to O_2_ stress for each culture. The sensitivity coefficients were determined to be 0.127 ± 0.004 and 0.086 ± 0.005 (± standard error) for the *T. elongatus* axenic (*TE*) and binary (Binary) cultures, respectively. Each data point represents the average of measurements taken every minute over a minimum of three reactor residence times held under each respective steady state. Error bars represent ±1 standard deviation. *, significant differences with >99% certainty as determined by *t* test of unequal sample sizes, assuming equal variance.

The binary consortium exhibited a decreased sensitivity to O_2_ stress compared to the *T. elongatus* axenic control (Fig. 2). A linear decrease in µ was observed, for both the axenic and binary cultures, as the partial pressure of O_2_ (pO_2_; corresponding to inlet gas composition) was raised from 0 through 0.59. This decrease was ameliorated in the binary culture. The maximum pO_2_ treatment provided a dissolved O_2_ concentration of 366 µM or 281% of air saturation (at 52°C). Oxygen sensitivities were measured as the absolute slope(s) 0.127 ± 0.004 and 0.086 ± 0.005 h atm-O_2_^−1^ (± standard error) for the *T. elongatus* axenic control and binary consortium, respectively.

### Cyanobacterial responses to heterotrophic partnership.

**FIG 3:**
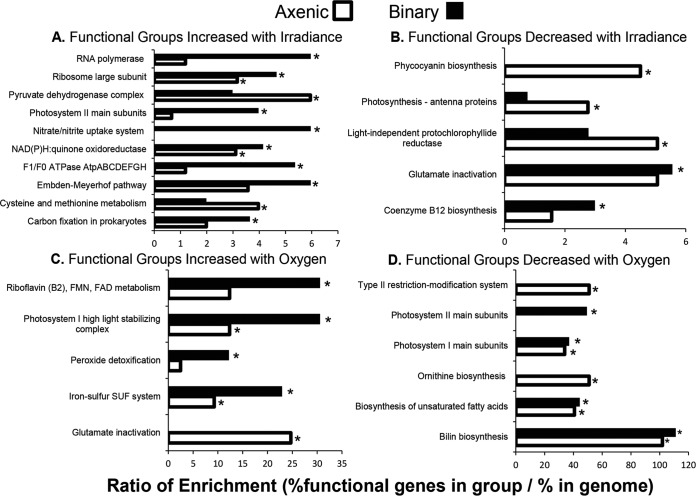
Enriched gene functions responding to irradiance and pO_2_. Ratios on the *x* axis depict the percentage of genes of a particular function (shown on the *y* axis) in a given category (e.g., RNA polymerase genes increasing their expression with irradiance under binary conditions, top black bar of the upper left figure) divided by the percentage of genes of the same function in the genome as a whole. For comparison, the ratios of functional enrichment are shown for both axenic (white) and binary (black) cultivation with at least one of the two being significantly enriched with a *P* value of <0.05 as determined by Fisher’s exact test (indicated by *). (A) Functional groups that increased with increasing irradiance. (B) Functional groups that decreased with increasing irradiance. (C) Functional groups that increased with increasing O_2_ tension (pO_2_); SUF, sulphur assimilation. (D) Functional groups that decreased with increasing O_2_ tension. FMN, flavin mononucleotide; FAD, flavin adenine dinucleotide.

*T. elongatus* showed a different transcriptional response in the presence of *M. ruber* than the axenic control. Light- and O_2_-responsive genes were defined as those showing ≥2-fold change between the lower and maximum experimental bounds with respect to I_i_ and pO_2_. Of the 2,476 protein-encoding genes in *T. elongatus*, 354 and 339 were determined to be light responsive under axenic and binary steady-state conditions (pO_2_ = 0 atm-O_2_), respectively. Similarly, 105 and 60 genes were determined to be O_2_ responsive under axenic and binary steady-state conditions (I_i_ = 1,995 µmol photons m^−2^ s^−1^), respectively. Different gene function categories were significantly enriched (*P* < 0.05) from groups determined to be either light or oxygen regulated (Fig. 3).

Genes whose transcripts were shown to be responsive by increasing with irradiance included those associated with photosystem II (PS II) (*psbV*, *psbX*, and *psbV2*; *tll1285*, *tsr2013*, and *tll1284*) and β-carboxysome (*ccmK1* and *ccmL*; *tll0946* and *tll0945*) functions. In addition to their response to I_i_, a greater number of PS II genes showed elevated transcript abundance during heterotrophic partnership (Fig. 3A). *T. elongatus* genes encoding significantly enriched functions related to pyruvate metabolism (*pdhB* and *pdhA*; *tll0204* and *tlr1169*) and metabolism of the amino acids cysteine and methionine (*cysE* and *metE*; *tlr0851* and *tlr1190*; *coaX* and *panC-cmk*; *tll1149* and *tll2450*) were light responsive with I_i_, but a smaller number of these genes exhibited such changes in expression as a result of *M. ruber* partnership compared to axenic growth (Fig. 3B).

The majority of functions involving O_2_-responsive genes showed higher enrichment ratios under binary than under axenic *T. elongatus* growth. These include PS I-stabilizing products, peroxide detoxification, and assembly of iron-sulfur clusters. The specific genes in these categories which showed increased expression with pO_2_ include photosystem-stabilizing genes *hliC* and *hliA* (*tsr0446* and *tsl2208*), iron sulfur genes *sufD* and *iscU* (*tlr1905* and *tll1093*), and peroxide detoxification genes *grxD* (*tll0874*) and *tll1454* (Fig. 3C). Among genes showing decreased expression with increasing O_2_, both type II restriction modification system gene *tll1944* and ornithine biosynthesis gene *argJ* (*tll1911*) were enriched only under axenic conditions.

**FIG 4:**
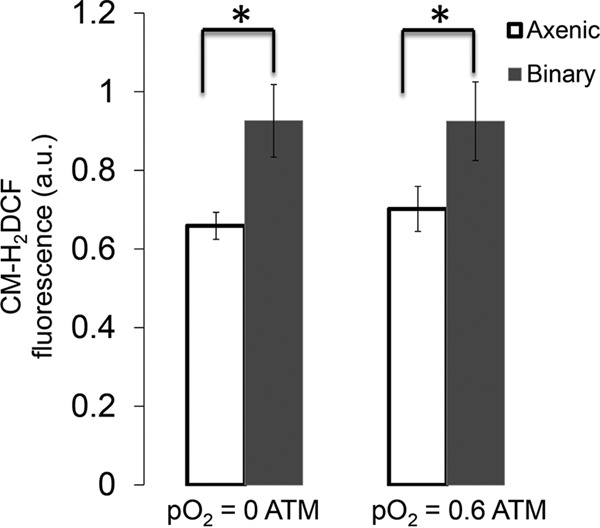
Intracellular reactive oxygen/nitrogen (ROS-RNS) increased under binary cultivation compared to axenic *T. elongatus* controls. Average intensity (*n* = 3) of the 525-nm fluorescence emitted from cells excited at 488 nm, corresponding to the abundance of oxidized ROS-RNS reporter dye (CM-H_2_DCFHDA) for the *T. elongatus* axenic and binary cultures maintained under low and high oxygen tensions. Error bars represent ±1 standard deviation. *, significant differences with >99% certainty as determined by Tukey’s test.

**FIG 5:**
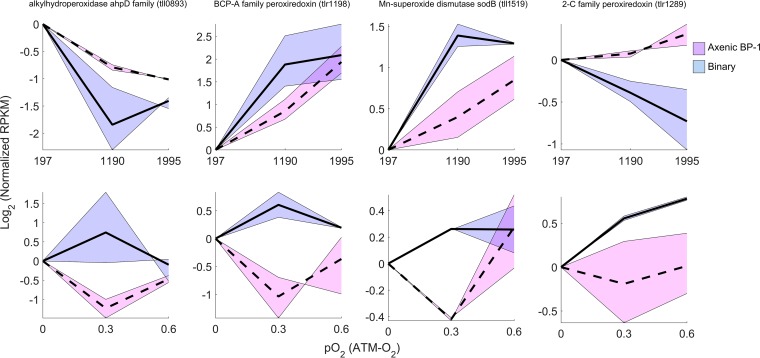
Relative abundance profiles for *T. elongatus* transcripts of ROS-RNS detoxification genes that showed at least a 1.5-fold change over either the irradiance- or pO_2_-controlled steady states. Solid black lines and shaded blue regions represent the means and ranges of *T. elongatus* genes expressed during binary cultivation. Similarly, dashed black lines and shaded red regions represent the means and ranges of *T. elongatus* genes expressed during axenic cultivation. The transcripts identified include a putative alkylhydroperoxidase *ahpD* family gene (*tll089*3), a BCP-A family peroxiredoxin (*tlr1198*), an Mn-superoxide dismutase (*sodB*; *tll1519*), and a 2-Cys family peroxiredoxin (*tlr1289*). “Normalized RPKM” is the average of duplicate RPKM values measured under each steady state divided by the average RPKM corresponding to the lowest incident irradiance (197 µmol photons m^−2^ s^−1^) or pO_2_ (0 atm-O_2_).

Another acclimation response to partnership was observed as increased relative intracellular abundances of reactive oxygen and nitrogen species (ROS and RNS, respectively) during binary cultivation compared to the *T. elongatus* axenic controls (Fig. 4). The fluorescence of an oxidized ROS-RNS reporter dye [5-(6)-chloromethyl-2′,7′-dichlorodihydrofluorescein diacetate, acetyl ester (CM-H_2_DCFHAD)] increased under binary cultivation but, unexpectedly, was insensitive to the increasing pO_2_ treatments. *T. elongatus* showed differential expression of ROS detoxification transcripts in the presence of *M. ruber* across a broad range of I_i_- and pO_2_-controlled steady states. In many cases, the expression patterns of these *T. elongatus* transcripts showed opposite trends between axenic and binary cultivation across the I_i_ and pO_2_ treatments (Fig. 5). Generally, an increased relative abundance of these transcripts was observed under binary cultivation compared to the *T. elongatus* axenic control. These results show that *T. elongatus* acclimates to partnership with *M. ruber* by changing the expression of ROS detoxification genes, which is likely driven by a heterotrophically mediated increase in ROS-RNS.

### Coordinated transcriptional patterns as a result of metabolic coupling.

**FIG 6:**
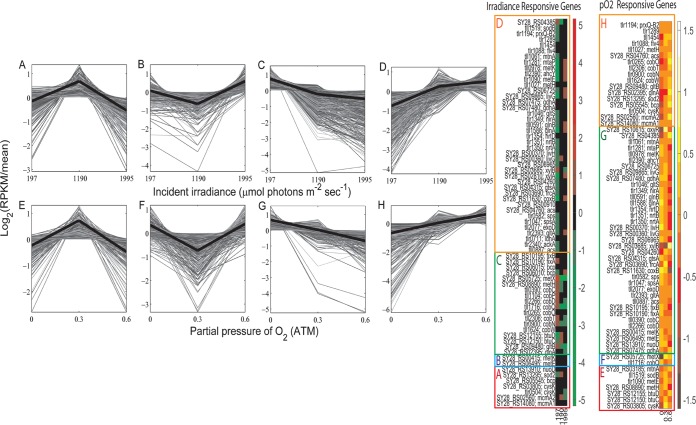
K-means clusters of *T. elongatus* and *M. ruber* relative mRNA abundance profiles that share correlated expression patterns over irradiance- and pO_2_-controlled steady states. Heavy black lines represent the centroid (i.e., eigen-gene) calculated within each cluster. Cluster A, 201 *T. elongatus* plus 259 *M. ruber* genes; cluster B, 255 *T. elongatus* plus 188 *M. ruber* genes; cluster C, 971 *T. elongatus* plus 444 *M. ruber* genes; cluster D, 914 *T. elongatus* plus 324 *M. ruber* genes; cluster E, 211 *T. elongatus* plus 328 *M. ruber* genes; cluster F, 187 *T. elongatus* plus 309 *M. ruber* genes; cluster G, 913 *T. elongatus* plus 468 *M. ruber* genes; cluster H, 888 *T. elongatus* plus 387 *M. ruber* genes. Heat maps show the expression patterns [log_2_(RPKM/mean)] for specific functional genes from each organism.

A guiding hypothesis was that since *T. elongatus* supported *M. ruber* via exchange of essential resources (i.e., C, N, and O_2_), *M. ruber* must coordinate its gene expression to accommodate the variable physiology of the cyanobacterium across the gradients of I_i_ and pO_2_ conditions. Examination of individual transcript profiles was performed by considering clusters around four main patterns specified by the centroid or “eigen-genes” corresponding to the I_i_ and pO_2_ treatments used to establish steady state (Fig. 6). Clusters A to D were calculated to examine responses to I_i_ treatments, and clusters E to H were calculated for examining the transcriptional effects from increasing O_2_ tension. All clusters contained both *T. elongatus* and *M. ruber* genes. Cluster A exhibits a tent-shaped eigen-gene with maximum relative mRNA abundances at the midpoint I_i_ (1,190 µmol photons m^−2^ s^−1^). The midpoint was chosen as a sampling condition because it is the theoretical saturating irradiance (26). Genes in cluster B responded inversely to those in cluster A. Clusters C and D contain genes that show a relative decrease or increase with I_i_, respectively. Note that the changes observed for the relative abundance of transcripts with increasing I_i_ also correspond to increasing specific growth and photosynthesis rates (Fig. 1). Clusters E to H contain transcripts that trend with increasing pO_2_ treatments (0 to 0.59 atm-O_2_) and show profiles that are analogous to clusters A to D. Changes observed for the relative abundance of transcripts with increasing pO_2_ correspond to a linear decrease in the specific growth rate (Fig. 2). Statistically enriched gene functions were identified for both *T. elongatus* and *M. ruber* within each cluster (Tables S1 and S2) to infer which processes may have been involved in metabolite exchange and acclimation to partnership.

10.1128/mSystems.00181-16.7 TABLE S1  Statistical/functional enrichment of *T. elongatus* gene functions within clusters of mRNA profiles. Download TABLE S1, PDF file, 0.2 MB. Copyright © 2017 Bernstein et al. 2017 Bernstein et al. This content is distributed under the terms of the Creative Commons Attribution 4.0 International license.

10.1128/mSystems.00181-16.8 TABLE S2  Statistical/functional enrichment of *M. ruber* gene functions within clusters of mRNA profiles. Download TABLE S2, PDF file, 0.2 MB. Copyright © 2017 Bernstein et al. 2017 Bernstein et al. This content is distributed under the terms of the Creative Commons Attribution 4.0 International license.

### Primary productivity and carbon exchange.

*T. elongatus* supported *M. ruber* growth via the production and transfer of reduced carbon. Cyanobacterial processes involved in biosynthesis and transport of organic carbon shared common transcriptional patterns with *M. ruber* genes encoding enzymes used in carbon uptake and metabolism of the same (or similar) gene products. Principal genes involved in organic acid synthesis of *T. elongatus* grouped into clusters that increased with I_i_ (cluster D) and/or decreased with pO_2_ (cluster G). These included acetyl coenzyme A (CoA) synthetase (*acs*; *tll0887*), acetate kinase (*ackA*; *tlr2340*), lactate dehydrogenase (*ldhA*; *tlr0711*), and citrate synthase (*gltA*; *tlr2393*). Functions involved in synthesis and export of larger biomolecules (i.e., sugars, peptides, and extracellular polymeric substance [EPS]) also grouped into clusters D and G. These included a putative exopolysaccharide synthesis gene (*exoD*; *tll2077*), sucrose synthase (*spsA*; *tlr1047*), and a sucrose degradation enzyme (*sps*; *tlr0582*). In conjunction with *T. elongatus*, *M. ruber* genes involved in the uptake and metabolism of compounds related to export and synthesis of *T. elongatus-*derived organic carbon were also found in clusters D and/or G. Notable examples included genes encoding putative acetyl-CoA synthetase (*acs*; *SY28_RS04760* and *SY28_RS00910*), cytochrome *c* oxidase (*coxB*; *SY28_RS11630*), monosaccharide uptake systems (*frcA* and *gtsAB*; *SY28_RS03690*, *SY28_RS04315*, and *SY28_RS04260*), xylose isomerase (*xylA*; *SY28_RS02810*), xylulokinase (*xylB*; *SY28_RS03685*), an ABC-type multisugar uptake system (*SY28_RS06965*), and branched-chain amino acid uptake (*livGH*; *SY28_RS00360* and *SY28_RS00370*).

### Nitrogen and amino acid exchange.

Since *M. ruber* lacks an assimilatory nitrate reductase, *T. elongatus* was assumed to provide reduced N during binary cultivation. The relative abundances of transcripts encoding the *T. elongatus* nitrate uptake system from *nrtABD* (*tlr1350*, *tlr1351*, and *tlr1354*) increased with I_i_, decreased with pO_2_, and grouped appropriately into clusters D and G. Other *T. elongatus* nitrogen metabolism genes found in these clusters include glutamine synthetase gene *glnA* (*tll1588*), a nitrogen regulatory protein gene (*glnB*; *tll0591*), an assimilatory ferredoxin-nitrate reductase gene (*nirA*; *tlr1349*), and a glutamate symporter gene (*gltS*; *tlr1046*). Several *M. ruber* nitrogen-associated genes also grouped into clusters D and/or G, including a glutamate dehydrogenase gene (*gdhA*; *SY28_RS07480* and *SY28_RS07475*) and amino acid uptake system genes (*SY28_RS09865* and *SY28_RS06725*). However, some key genes required for N acquisition by *M. ruber* grouped into clusters C and/or H, showed opposite expression patterns with respect to I_i_ and pO_2_, and effectively increased with the specific growth and photosynthesis rates. These include *glnA* (*SY28_RS02395*) and the large subunit of glutamate synthase (*gltB*; *SY28_RS09480*) and suggest the potential for direct exchange of glutamine and glutamate from *T. elongatus* as growth requirements increase with the specific growth and photosynthesis rates.

### Methionine and vitamin B_12_ exchange.

Cobalamin (B_12_) auxotrophy in *M. ruber* is indicated by the absence of a complete B_12_ synthesis pathway and a B_12_-independent methylcitrate pathway (*prpBCDF*) required for conversion of propionyl-CoA to succinyl-CoA. *M. ruber* contains the vitamin B_12_-dependent methylmalonyl-CoA mutase subunits (*mcmA1* and -*A2*; *SY28_RS14080* and *SY28_RS02560*) which grouped into clusters A and H. *T. elongatus* is a B_12_ prototroph, as confirmed by the capacity for axenic growth in the absence of vitamin B_12_ and by the presence of a complete vitamin B_12_ biosynthesis pathway in the genome. The relative abundances of transcripts encoding vitamin B_12_ uptake/scavenging gene products in *M. ruber* (*btuCD*; *SY28_RS12150* and *SY28_RS12155*) decreased with I_i_ in concurrence with decreased *T. elongatus* transcripts (all within cluster C) encoding vitamin B_12_ biosynthesis, including those required for the insertion of cobalt (*cobWNT*; *tll1624*, *tlr0900*, and *tll2306*) and for the conversion of cobyrinic acid diamine to the vitamin B_12_ coenzyme from *cobOQDPC* (*tlr0265*, *tll1716*, *tll2266*, *tll1104*, and *tll0390*). Both *T. elongatus* and *M. ruber* expressed transcripts encoding the vitamin B_12_-dependent homocysteine methyltransferase (*metH*) but were negatively correlated and grouped across I_i_ and pO_2_ treatments into opposing clusters D (*tll1027*) and C (*SY28_RS08890*), indicating that methionine may be directly exchanged from *T. elongatus* as growth requirements increase with the specific growth rate. Both strains also expressed vitamin B_12_-independent homocysteine methyltransferase (*metE*) transcripts which grouped into clusters D and E compared to clusters B and G for *tlr1090* and *SY28_RS06495*, respectively.

The relative abundances of *T. elongatus* transcripts encoding the degradation and salvage of methionine (*ahcY*, *metK*, *mtaP*, and *mtnA*; *tll2390*, *tll0978*, *tlr1281*, and *tll1061*) increased with I_i_ but decreased with pO_2_ treatments and grouped within clusters D and G, respectively. Uniform grouping of *M. ruber* transcripts encoding methionine degradation and salvage was not observed as they were spread across multiple clusters grouped by the I_i_ and pO_2_ treatments: *metK* (*SY28_RS00415*; clusters B and G) and *mtnA* (*SY28_RS03185*; cluster E). In contrast, the relative abundance of *M. ruber* transcripts encoding methionine biosynthesis proteins decreased with I_i_, and they were grouped into cluster C, including *metHX* (*SY28_RS08890* and *SY28_RS05725*). Genes involved in cysteine biosynthesis shared common transcriptional patterning between species, indicating that while *M. ruber* may have salvaged cyanobacterium-derived methionine, it likely synthesized its own cysteine via the vitamin B_12_-independent pathway as growth requirements increased. These include cysteine synthases (*cysK*; *tlr0504* and *SY28_RS03805*) and serine O-acetyltransferases (*cysE*; *tlr0851* and *SY28_RS05065*), which cogrouped into clusters D and A, respectively.

### Oxidative stress responses.

The relative abundance of *T. elongatus* transcripts encoding enzymes involved with ROS detoxification generally increased with increasing I_i_ and pO_2_, and the transcripts grouped into the appropriate clusters D and/or H. Notable examples include an NAD(P)H-oxygen oxidoreductase (*flv4*; *tlr1088*), 2-Cys family peroxiredoxins (*tll1454* and *tlr1289*), a periplasmic peroxiredoxin (*prxQ-B2*; *tlr1194*), and an Mn-superoxide dismutase (*sodB*; *tll1519*). *M. ruber* contains an H_2_O_2_-responsive transcriptional regulator (*oxyR*; *SY28_RS10615*), which grouped into clusters D and G and is located adjacent to a putative manganese catalase (*SY28_RS10610*). However, *M. ruber* peroxidase (*bcp*; *SY28_RS05545*, *SY28_RS06010*, and *SY28_RS06015*) and superoxide dismutase (*sod2*; *SY28_RS13295*) genes responded differently to I_i_ treatments than did *oxyR* and related cyanobacterial profiles and were grouped into clusters A and C. These genes generally increased with pO_2_ and grouped with the *T. elongatus* genes into cluster H (increased with pO_2_ and decreasing µ). *M. ruber* genes associated with electron transfer processes that are potentiators of ROS (27, 28) grouped into cluster G, which decreased with increasing pO_2_ treatments and increased with specific growth and photosynthesis rates. These include subunits for an electron transfer flavoprotein (*fixAB*; *SY28_RS10190* and *SY28_RS10195*), NADH-dehydrogenase (*SY28_RS04385*), and principal components of the NADH-quinone oxidoreductase (*nuoDFGHIJKN*).

## DISCUSSION

The cyanobacterium *T. elongatus* responded to heterotrophic partnership with *M. ruber* by altering the expression of key functional genes. This primary result is evidence of indirect interspecies regulation. It is important to note that the turbidostat culturing platform provided a constant, optically thin, nutrient-replete environment. Hence, the addition of *M. ruber* to *T. elongatus* cultures did not alter the growth environment by reducing availability of actinic light or nutrients needed to support *T. elongatus*. The net specific rate of O_2_ production decreased during binary cultivation compared to the *T. elongatus* axenic controls. These rates are functionally equivalent to net photosynthesis rates and account for the gross rate of oxygenic photosynthesis minus all oxygen-consuming reactions, including photorespiration (RuBisCO oxygenase activity) and heterotrophic respiration (29, 30). The net photosynthesis rates are conservative interpretations for the lower bound of oxygenic photosynthesis and relatable as proxy measurements for the minimum consortium-wide energy acquisition rates, assuming 0.125 quanta absorbed and 2 NADPH produced per mole O_2_ formed via PS II-mediated charge separation (31). Hence, the energy efficiency for biomass production is greater in the binary consortium than in the *T. elongatus* axenic control. These increases likely result from heterotrophic capture of reductant that would otherwise be lost from the system as either photosynthate or necromass. The addition of *M. ruber* partnership also resulted in an observed decrease in O_2_ sensitivity compared to *T. elongatus* axenic controls (Fig. 2). A decrease in sensitivity is equivalent to an increase in O_2_ stress resistance. We note that this effect was observed under identical pO_2_ treatments (binary versus axenic), which supported O_2_ tensions sufficient to render the bulk effects resulting from heterotrophic O_2_ removal as negligible. However, attachment or close cellular proximity of *M. ruber* to *T. elongatus* cells may create localized gradients and microheterogeneities in I_i_ and pO_2_ experienced by the *T. elongatus* population. Interactions that occur when cells from each species are in close contact could account for the observed differences in O_2_ sensitivity and the photosynthetic quotients which were based on measurements taken from the well-mixed (bulk) volume.

The *M. ruber*-induced decrease of O_2_ sensitivity (Fig. 2) originally led us to hypothesize that heterotrophic partnership reduced cyanobacterial oxidative stress and that this effect could be observed by comparing intracellular ROS-RNS between axenic and binary conditions. Interestingly, we found that heterotrophic partnership had the opposite effect and that increased pO_2_ had no effect on intracellular ROS-RNS (Fig. 4). However, the binary culture’s observed decrease in O_2_ sensitivity did correspond to enrichment of genes within the functional category photosystem I high-light-stabilizing complex (Fig. 3). The *T. elongatus* genes in this category include *hliACD* (*tsl2208*, *tsr0446*, and *tsr1916*) and *tsl0063*, annotated as a member of the CAB/ELIP/HLIP protein family, which are known to be stress-induced genes that help cyanobacteria cope with free radicals and excess excitation energy (32, 33). The increases in ROS-RNS observed as a direct result of heterotrophic partnership corresponded with increased relative transcript abundances of *T. elongatus* genes required for mitigating ROS, showing that the cyanobacterium acclimates to heterotrophic partnership by increasing protection from oxidative stress (Fig. 4 and 5). This is contrary to previous observations made in consortia constructed from oligotrophic cyanobacteria and heterotrophs, in which *Prochlorococcus* species have been reported to benefit and/or depend upon heterotrophic bacteria, such as *Alteromonas*-like species, to reduce oxidative stress (34, 35). In contrast, evidence of cyanobacterium-mediated ROS mitigation was reported within the binary consortium of *Synechococcus* sp. strain PCC 7002 coupled with *Shewanella putrefaciens* W3-18-1, cultured under very different conditions than those employed in the current study (2). Both *T. elongatus* and *Synechococcus* sp. PCC 7002 were isolated from eutrophic environments (mat and marine sediment, respectively), which can presumably support higher levels of heterotrophic growth than oligotrophic marine environments. Cyanobacteria are equipped to detoxify intracellular ROS-RNS and mitigate oxidative stress, because ROS production is an inherent by-product of oxygenic photosynthesis and photosynthetic electron transfer (36, 37), while heterotrophic bacteria are recognized for production of extracellular ROS (27). The principle that is inferred from these collective results is that some cyanobacteria, such as *Synechococcus* species adapted to eutrophic environments exposed to high irradiance, specialize in ROS-RNS mitigation (38, 39).

The metabolic dependency of *M. ruber* upon *T. elongatus* for reduced carbon, nitrogen, and certain vitamins (i.e., B_12_, biotin, and niacin) was corroborated through the coexpression of genes encoding enzymes that metabolize and transport shared metabolites. These inferences are made through the basic assumption that the coexpressed genes, i.e., correlated mRNA abundances between the two species, correspond to protein frequency and activity. Clustering the relative mRNA abundance profiles of the two organisms together provided the means to infer which specific metabolic exchanges are coordinated and how these interactions respond to I_i_ and pO_2_. Specifically, the results suggest that multiple carbon compounds derived from the cyanobacterium could be exchanged and taken up by *M. ruber*. While evidence for the exchange of organic acids (e.g., acetate) was observed, polysaccharides, peptides, and EPS may have also supplied the carbon and reductant required to support stable *M. ruber* populations. Ample evidence also supports the likelihood that amino acids can serve as a source of reduced N (and possibly carbon) required by *M. ruber*. For example, the expression of genes involved in the synthesis, transport, and salvage of specific amino acids showed coordinated patterns shared by each species. Specifically, *T. elongatus* transcripts of methionine and glutamate biosynthesis genes clustered into groups that increased with µ (clusters D and G) while *M. ruber* homologs showed opposite patterns (clusters C and H), suggesting the specific exchange of these amino acids. The relative abundance profiles for *M. ruber* genes encoding vitamin B_12_-dependent methionine synthesis showed expression patterns in common with vitamin B_12_ salvage and *T. elongatus* vitamin B_12_ synthesis genes, indicating that one exchanged resource (e.g., vitamin B_12_) may affect requirement of another exchangeable resource (e.g., methionine) that has a closely linked pathway.

**FIG 7:**
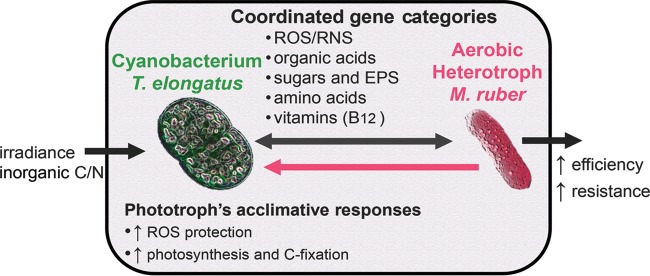
Schematic outline of responses to heterotrophic partnership observed and inferred as indirect interspecies regulation events and metabolic coupling.

The collection of experimental results presented here clearly shows that the cyanobacterium responded and acclimated to heterotrophic partnership. In this experimental system, *T. elongatus* is either indirectly regulated by environmental changes instigated by *M. ruber* or directly regulated via molecular signals. The integrated kinetics- and global transcriptomics-based inferences were not targeted enough to capture a mechanism for direct interspecies regulation. Although direct interspecies regulation cannot be ruled out, our conclusion is that this heterotrophic partnership establishes an indirect interspecies regulation of gene expression resulting in measurable changes of growth and photosynthesis kinetics of the binary culture compared to the cyanobacterial axenic control. The outcomes described here can be generalized to understand microbial cyanobacterium-heterotroph interactions better (Fig. 7). For instance, the cyanobacterium was inferred to sense the presence of its commensal heterotrophic partner and respond by altering its gene expression. Interspecies modulation of gene expression, via indirect regulation, is supported by results reported in previous studies that investigated very different cyanobacterium-heterotroph platforms (2, 4), and a number of notable commonalities emerge. A previous study, investigating a consortium composed of cyanobacterium *Synechococcus* sp. PCC 7002 coupled with *Shewanella putrefaciens* W3-18-1 (2), reported a >2-fold change in mRNA abundances of cyanobacterial genes (compared to axenic) that encode enzymes belonging to the same functional groups that were statistically enriched from *T. elongatus* in the current study (Fig. 3). These include some of the most notable examples, such as carbon concentration, carboxysome synthesis, PS II, oxidative phosphorylation, methionine metabolism, and Fe-S cluster biogenesis. Similarly to the results from this study, *Synechococcus* sp. PCC 7002 was also reported to show >2-fold changes in B_12_ salvage processes when partnered with *S. putrefaciens* W3-18-1, although the current study investigated a cyanobacterial B_12_ prototroph. Another study, which investigated the transcriptional responses of *Synechococcus* sp. WH8102 cocultured with *Vibrio parahaemolyticus*, also reported changes in expression of genes sharing the functions reported here. These include amino acid biosynthesis, cofactor biosynthesis, PS II, NAD(P)H-dehydrogenase, and ATPase. The functional similarities of cyanobacterial genes being influenced and potentially indirectly regulated by the activity of a heterotrophic partner are remarkable considering the difference in species, their origins, and the treatments and culturing platforms compared. Interspecies modulation of gene expression likely serves as a fundamental principle enabling microbial communities to coordinate their metabolism and coacclimate to each other and environmental cues.

## MATERIALS AND METHODS

### Bacterial strains and culturing media.

Both *Thermosynechococcus elongatus* strain BP-1 and *Meiothermus ruber* strain A were grown axenically and as a binary consortium in a modified BG-11 (mBG-11) medium (BP-1 medium) containing 17.6 mM NaNO_3_, 0.304 mM MgSO_4_⋅7H_2_O, 0.175 mM KH_2_PO_4_, 0.245 mM CaCl_2_⋅2H_2_O, 0.0028 mM Na_2_EDTA, and 0.0144 mM FeCl_3_. The mBG-11 was supplemented with 1 ml liter^−1^ trace mix: 46.2544 mM H_3_BO_3_, 9.1458 mM MnCl_2_⋅4H_2_O, 0.772 mM ZnSO_4_⋅7H_2_O, 1.611 mM Na_2_MoO_4_⋅2H_2_O, 0.316 mM CuSO_4_⋅5H_2_O, and 0.170 mM Co(NO_3_)_2_⋅6H_2_O. During binary culture, both species were supported by the autotrophic growth of *T. elongatus* (i.e., no organic substrate was added for the growth of *M. ruber*). Axenic starter cultures of *M. ruber* were cultured in mBG-11 supplemented with 1% yeast extract. Starter cultures were generated in 150-ml sealed serum bottles charged with 50 ml mBG-11 plus 15 mM NaHCO_3_ (adjusted to pH 7.5) under 10% CO_2_ in N_2_ headspace. Photobioreactors were inoculated to an optical density at 730 nm (OD_730_) of 0.01 with exponentially growing starter culture(s).

### Turbidostat photobioreactor operation.

This study modified a previously reported continuous stirred tank reactor operated with feedback control as a turbidostat (40–43). Briefly, a “white-light” photobioreactor (7.5-liter vessel) was constructed with evenly spaced sets of fluorescent lights (Sylvania; model FP14/835/ECO, Pentron 3500 K, 14 W) at each quadrant of the cylindrical bioreactor vessel (both 8- and 12-bulb configurations). A custom dimmer was controlled with a 4- to 20-mA signal commanded from a New Brunswick Scientific BioFlo 310 bioreactor controller. The reactor and light bank apparatus were isolated from external ambient light. The working volume of the reactor (5.5 liters) was held at a constant temperature of 52°C and pH 7.5. A Clark-type oxygen sensor coupled with two independent off-gas sensors (Blue Sense; Cell and Ferm models) was used to measure dO_2_ and off-gas O_2_ and CO_2_, respectively. Incident scalar irradiance and the transmitted irradiance used to control turbidostat operation were maintained using custom photovoltaic cells coupled with a digital pA/mV multimeter (Keithley 2700 Integra Series; Tektronix, Beaverton, OR). The dilution rate of the culture was determined gravimetrically from the effluent medium. Steady states were defined by stable (<10% variation from the mean) measurements of dilution rate, OD_730_, and dissolved O_2_ for a minimum of 3 residence times. These measurements, as well as off-gas CO_2_, pH, temperature, and acid/base addition, were data logged at 1-min intervals; hence, the replication of values used to calculate the specific rates (presented in Fig. 1 and 2) is means and standard deviations from a minimum of 604 measurements (for the fastest residence time of 3.36 h; dilution). Additional details for bioreactor-enabled measurements are available in Text S1 in the supplemental material.

10.1128/mSystems.00181-16.1 TEXT S1  Supplemental text describing additional details pertaining to the methodology and results presented in the manuscript and supplemental files. Download TEXT S1, DOCX file, 0.04 MB. Copyright © 2017 Bernstein et al. 2017 Bernstein et al. This content is distributed under the terms of the Creative Commons Attribution 4.0 International license.

### Flow cytometry.

The flow cytometry data were obtained using a BD Influx fluorescence-activated cell sorter (FACS; BD Biosciences, San Jose, CA). Using the 488-nm excitation from a Sapphire LP laser (Coherent Inc., Santa Clara, CA) at 100 mW, samples were analyzed using a 70-μm nozzle. Optimization and calibration of the FACS were performed before each analysis using 3-μm Ultra Rainbow fluorescent particles (Spherotech, Lake Forest, IL). The ratio of the two distinct populations of cells within a mixed microbial community was identified from 50,000 recorded cells using size and complexity gates with FCS Express (Los Angeles, CA) flow cytometry software. Cell counts are presented as the percentage of total counting events.

### RNA isolation and sequencing.

Cells for total RNA isolation were harvested from steady-state growth conditions by previously reported methods (2, 43). Transcriptomic samples were sampled in a minimum of two biological duplicates sampled from steady states in a minimum of 1 residence time (1/dilution rate) apart from each other. Briefly, cells were collected via centrifugation at 7,000 rpm for 5 min at 4°C, frozen in liquid nitrogen, and stored at −80°C. All RNA analyses were repeated in biological duplicates. To analyze the response of *T. elongatus* alone to the addition of a heterotrophic partner, RNA was extracted with TRIzol and sequenced using SOLiD (sequencing by oligonucleotide ligation and detection) technology with no depletion of rRNA (see the supplemental material for details). However, as binary culture samples contained <10% *M. ruber* by cell count, the extraction and sequencing methodology was modified when collecting data used to specifically examine the correlation of transcriptional responses between *T. elongatus* and *M. ruber*. For this, RNA was extracted using the SV total RNA isolation system (Promega, Madison, WI) followed by genomic DNA removal and purification using the Turbo DNA-free kit (Life Technologies, Carlsbad, CA). An Agilent 2100 Bioanalyzer was used to assess the integrity of the RNA samples. Only RNA samples having an RNA integrity number between 8 and 10 were used. cDNA libraries were constructed using the Ovation Universal transcriptome sequencing (RNA-seq) system (NuGEN, San Carlos, CA). This kit was used to incorporate specific InDA-C probes used to deplete rRNA from the binary culture experiments. Probes were designed to deplete both 16S and 23S rRNA from both *T. elongatus* and *M. ruber*. cDNA libraries were examined using the Agilent 2100 Bioanalyzer to confirm proper size and construction and were sequenced on an Illumina NextSeq 500 sequencer. Reads from all samples were aligned to the genomes of *T. elongatus* BP-1 (NCBI accession no. NC_004113) and the RAST gene model of *Meiothermus ruber* strain A (NCBI accession no. NZ_JXOP01000000) using the Burrows-Wheeler Aligner (BWA) with the default settings (44). Gene counts were determined using HTSeq (45) and were normalized first with DESeq2 (46) followed by normalization to the gene length in kilobases. Additional details on RNA sequencing and transcriptomic data analyses are available in the supplemental material.

### Transcriptomic analyses.

mRNA abundance profiles (given in reads per kilobase per million [RPKM]) for each steady-state condition (measured in biological duplicate) were filtered to remove any gene with an average count of zero in any condition and any gene with an average RPKM of <15. Binary culture expression profiles (genes from both organisms) were grouped via correlation-based K-means clustering (see the supplemental material). Prior to clustering, filtering was employed to mask the bottom 30% of genes with the smallest variance across each respective profile (i.e., flat profiles). Genes were determined to be significantly different in their expression when comparing two conditions if they showed a >2.0-fold change in their expression with an adjusted *P* value of <0.05. Significant enrichment (or functional enrichment) was performed as previously described (43). Briefly, enrichment was defined as the percentage of genes of a given function in the profile depicted (for example, increased expression with increased irradiance under binary conditions) divided by the percentage of genes in that same function in the genome as a whole. Enrichment ratios were then determined to be statistically significant using Fisher’s exact test with any ratio with a *P* value of <0.05 classed as significant.

### ROS-RNS measurements.

Relative abundances of reactive oxygen and nitrogen species (ROS-RNS) were measured using a previously reported method (47). Briefly, we used the cell-permeant fluorescent dye 5-(6)-chloromethyl-2′,7′-dichlorodihydrofluorescein diacetate, acetyl ester (CM-H_2_DCFHDA; Cayman Chemicals, Ann Arbor, MI), for which increased dichlorofluorescein (DCF) fluorescence is a proxy measurement of reactive oxygen/nitrogen species; increased fluorescence is proportional to increased intracellular ROS-RNS. Cell suspensions were collected from a steady-state culture and analyzed in triplicate (3 subfractions of cells collected from each steady state). For each replicate, a 1-ml aliquot was treated with 10 mM CM-H_2_DCFHDA freshly dissolved in 100% Me_2_SO (Sigma-Aldrich, St. Louis, MO) to a final concentration of 10 μM. Concurrently, a 1-ml aliquot of control cells was treated with 1 µl of Me_2_SO. Cells were transferred to opaque black 96-well plates (Nunc A/S, Roskilde, Denmark) and incubated in the dark with intermittent shaking for 30 min at room temperature. Fluorescence was measured at 525 nm after excitation at 488 nm on a SpectraMax Gemini XS spectrofluorometer. The control measurements were subtracted from the CM-H_2_DCFHDA-treated samples to correct for nonspecific fluorescence.

10.1128/mSystems.00181-16.9 DATA SET S1  Axenic *T. elongatus* transcript abundances and gene annotations. Download DATA SET S1, XLSX file, 0.8 MB. Copyright © 2017 Bernstein et al. 2017 Bernstein et al. This content is distributed under the terms of the Creative Commons Attribution 4.0 International license.

10.1128/mSystems.00181-16.10 DATA SET S2  Binary culture, *T. elongatus* and *M. ruber*, transcript abundances, gene annotations, and clustering assignments. Download DATA SET S2, XLSX file, 4.4 MB. Copyright © 2017 Bernstein et al. 2017 Bernstein et al. This content is distributed under the terms of the Creative Commons Attribution 4.0 International license.

### Accession number(s).

Raw sequencing files are available at the NCBI-GEO repository under accession numbers GSE94125 and GSE93859.
