# Supplementary material for: Indirect Interspecies Regulation: Transcriptional and Physiological Responses of a Cyanobacterium to Heterotrophic Partnership
Source: mSystems. 2017 Mar 7;2(2):e00181-16. doi: 10.1128/mSystems.00181-16 (PMC5340862; doi:10.1128/mSystems.00181-16)
Supplement: FIG S1 [file sys002172092sf4.pdf]

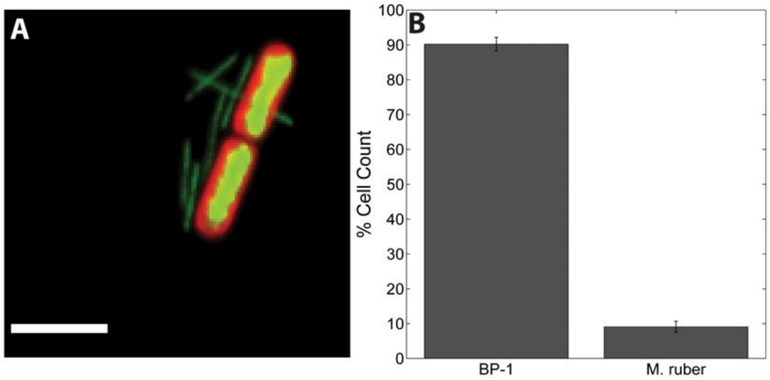

Figure S1. A) A representative confocal micrograph (1 of 40) showing cells maintained as a stable, photosynthetically supported binary culture of *T. elongatus* (red; autofluorescence) supporting *M. ruber* (green; SYBR Gold). Scale bar represents 10 µm. B) Relative abundances measured in % cells counted via fluorescence activated cell sorting. Values represent the mean from three independent steady states held under different oxygen tensions (pO_2 = 0, 0.3 and 0.6 ATM-O_2). Error bars represent ± 1 standard deviation.
