# Supplementary material for: Indirect Interspecies Regulation: Transcriptional and Physiological Responses of a Cyanobacterium to Heterotrophic Partnership
Source: mSystems. 2017 Mar 7;2(2):e00181-16. doi: 10.1128/mSystems.00181-16 (PMC5340862; doi:10.1128/mSystems.00181-16)
Supplement: FIG S2 [file sys002172092sf5.pdf]

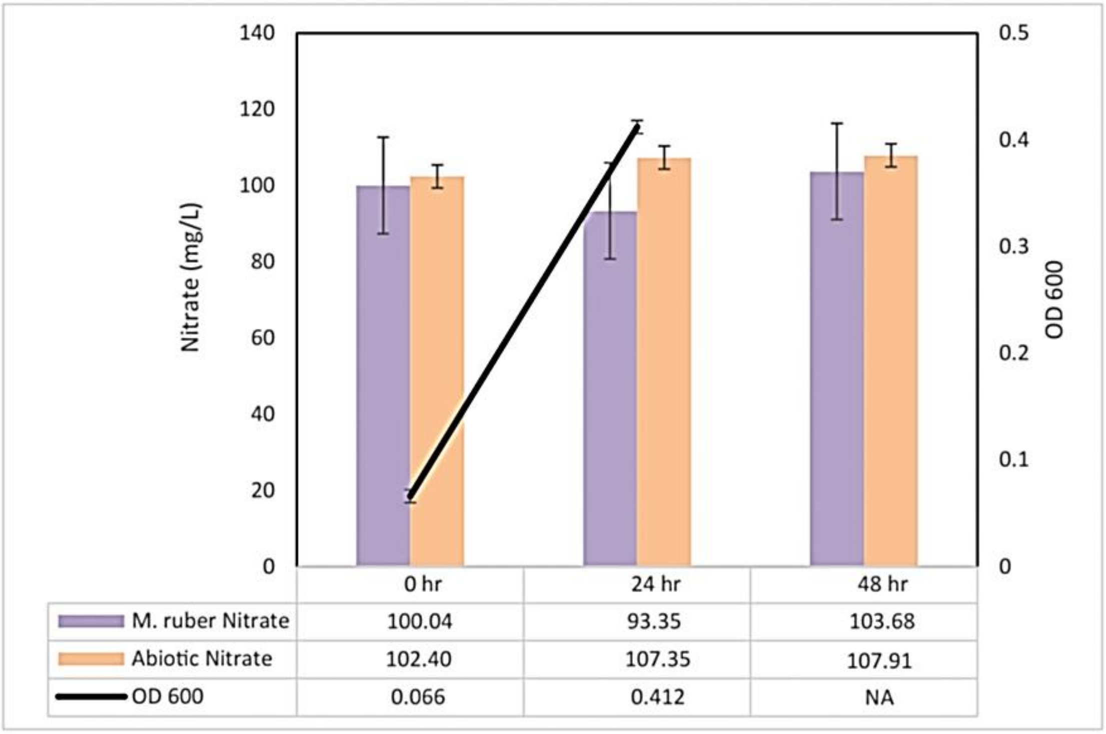


**Figure S2**. Batch growth and nitrate assay. Each data point represents the mean from three biological replicates. The optical density (OD 600) of *M. ruber* cells are matched to the *M. ruber* Nitrate samples. Error bars represent ± 1 standard deviation. The abiotic control experiment revealed no change in nitrate concentration and remained constant at 106 ± 3 mg $L^{-1}$ over 48 h.
