# Supplementary material for: Indirect Interspecies Regulation: Transcriptional and Physiological Responses of a Cyanobacterium to Heterotrophic Partnership
Source: mSystems. 2017 Mar 7;2(2):e00181-16. doi: 10.1128/mSystems.00181-16 (PMC5340862; doi:10.1128/mSystems.00181-16)
Supplement: FIG S3 [file sys002172092sf6.pdf]

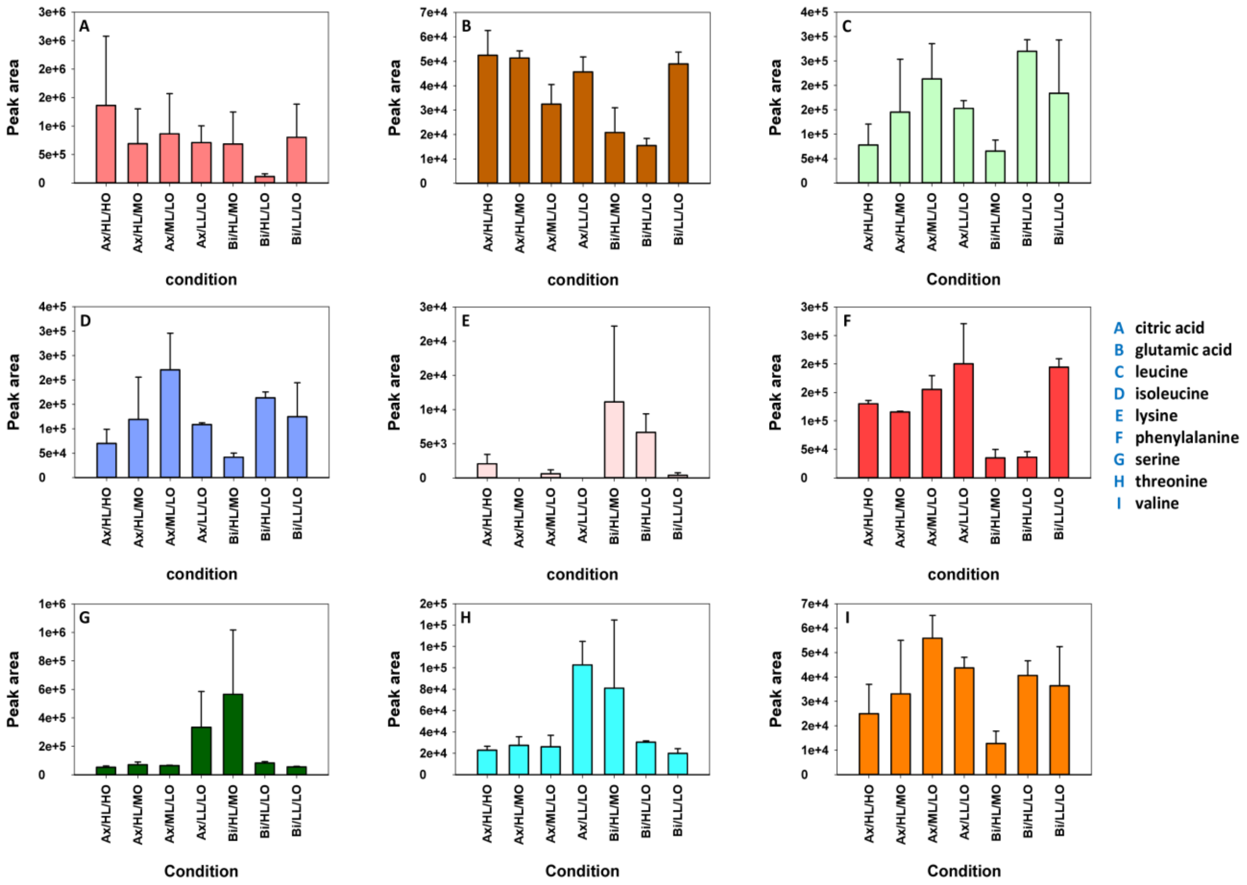

Figure S3. Extracellular metabolite measurements of axenic *T. elongatus* (Ax) and the binary culture (Bi). Specific turbidostat steady-state conditions are designated with the following abbreviations: high light (1995 µmol photons m^{-2} s^{-1}; HL), medium light (1190 µmol photons m^{-2} s^{-1}; ML), low ight (197 µmol photons m^{-2} s^{-1}; LL), high O_2 (pO_2=0.6 ATM; HO), medium O_2 (pO_2=0.3 ATM; MO) and low O_2 (pO_2=0.0 ATM; MO). Measurements are the mean from two biological replicates with standard error.
