# Supplementary material for: Indirect Interspecies Regulation: Transcriptional and Physiological Responses of a Cyanobacterium to Heterotrophic Partnership
Source: mSystems. 2017 Mar 7;2(2):e00181-16. doi: 10.1128/mSystems.00181-16 (PMC5340862; doi:10.1128/mSystems.00181-16)
Supplement: FIG S4 [file sys002172092sf7.pdf]

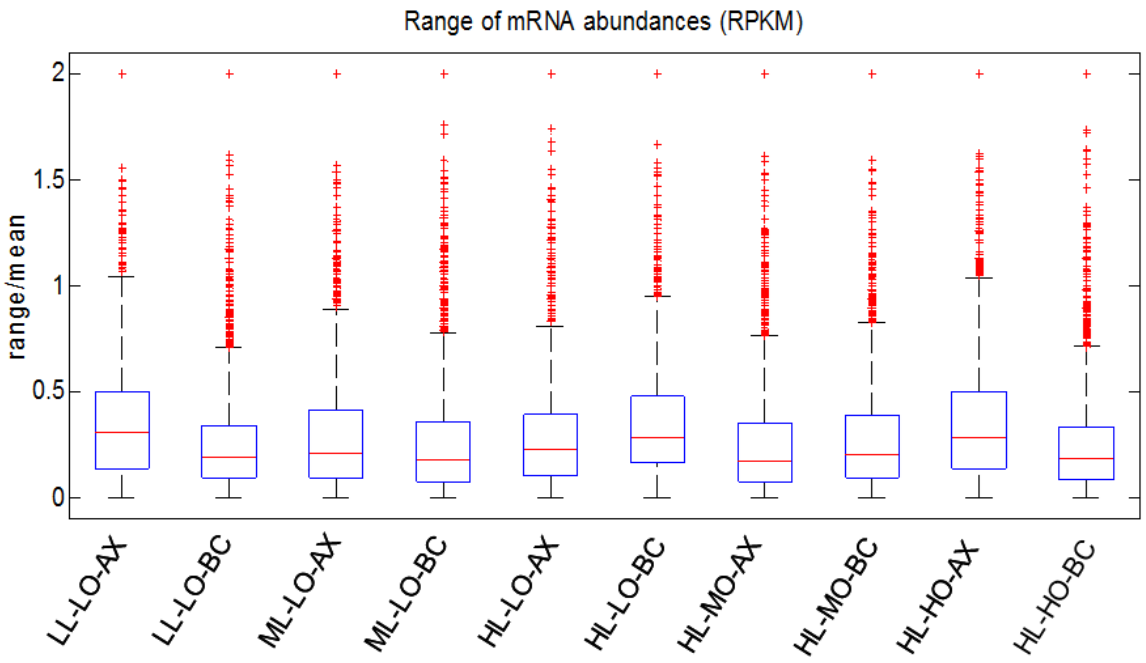

Figure S4. Concordance of replication shown as the ranges between biological duplicate measurements of mRNA abundances (RPKM) values for 2479 genes at each steady-state condition for which *T. elongatus* gene expression was analyzed. Specific turbidostat steady-state conditions are designated with the following abbreviations: axenic *T. elongatus* (AX), binary culture (BC), high light (1995 μmol photons m^{-2} s^{-1}; HL), medium light (1190 μmol photons m^{-2} s^{-1}; ML), low light (197 μmol photons m^{-2} s^{-1}; LL), high O_2 (pO_2=0.6 ATM; HO), medium O_2 (pO_2=0.3 ATM; MO) and low O_2 (pO_2=0.0 ATM; MO).
