# Supplementary material for: Indirect Interspecies Regulation: Transcriptional and Physiological Responses of a Cyanobacterium to Heterotrophic Partnership
Source: mSystems. 2017 Mar 7;2(2):e00181-16. doi: 10.1128/mSystems.00181-16 (PMC5340862; doi:10.1128/mSystems.00181-16)
Supplement: FIG S5 [file sys002172092sf8.pdf]

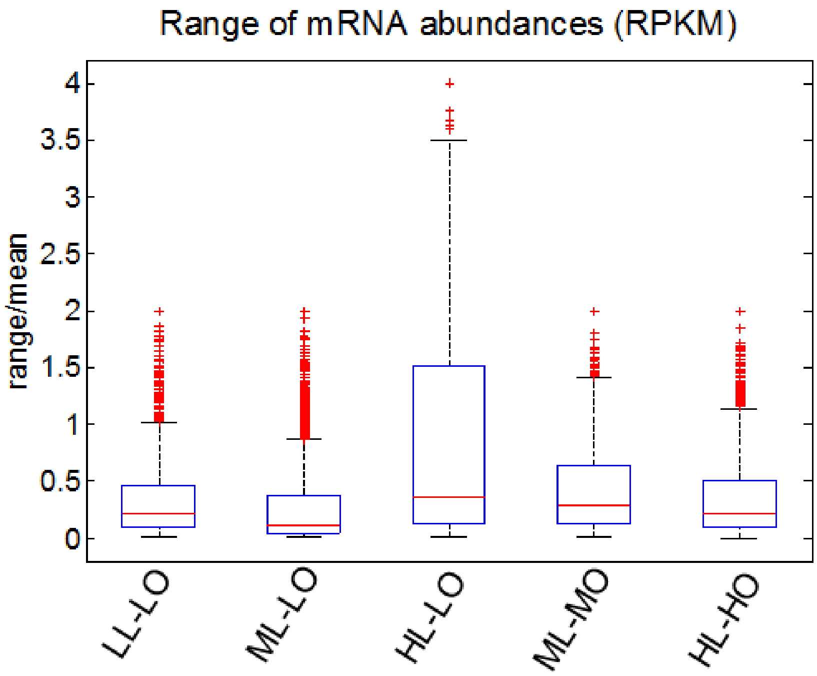

Figure S5. Concordance of replication shown as the ranges between biological replicate measurements of mRNA abundances (RPKM) values for 4492 genes at each steady-state condition for which species resolved gene expression was analyzed; binary cultivation of *T. elongatus* and *M. ruber*. Specific turbidostat steady-state conditions are designated with the following abbreviations: high-light (1995 µmol photons m^{-2} s^{-1}; HL), medium-light (1190 µmol photons m^{-2} s^{-1}; ML), low-light (197 µmol photons m^{-2} s^{-1}; LL), high-O_2 (pO_2=0.6 ATM; HO), medium-O_2 (pO_2=0.3 ATM; MO) and low-O_2 (pO_2=0.0 ATM; MO). Replication of transcriptomic analyses was performed in duplicate with the exception of condition HL-LO which was performed in quadruplicate.
