# Supplementary material for: Indirect Interspecies Regulation: Transcriptional and Physiological Responses of a Cyanobacterium to Heterotrophic Partnership
Source: mSystems. 2017 Mar 7;2(2):e00181-16. doi: 10.1128/mSystems.00181-16 (PMC5340862; doi:10.1128/mSystems.00181-16)
Supplement: TABLE S1 [file sys002172092st9.pdf]

Table S1. Statistical/functional enrichment of *T. elongatus* gene functions within clusters of mRNA profiles.

Subsystem	Cluster	Eigen-Gene	p-value	% in Module	% in Genome	Ratio
allophycocyanin biosynthesis	A-light	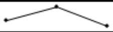	0.026	1.493	0.245	6.1
cytochrome c oxidase CoxABCD	A-light	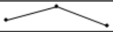	0.049	0.995	0.123	8.1
glycine cleavage system GcvHLPT	A-light	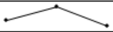	0.049	0.995	0.123	8.1
photosystem II other common subunits	A-light	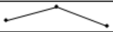	0.035	1.990	0.532	3.7
RNA polymerase sigma factors	A-light	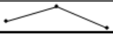	0.045	1.493	0.327	4.6
double strand breaks repair	B-light	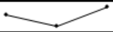	0.021	1.569	0.327	4.8
photorespiration (oxidative C2 cycle)	B-light	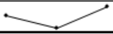	0.047	0.784	0.082	9.6
coenzyme A biosynthesis from pantothenate	C-light	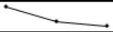	0.045	0.713	0.164	4.4
Uncharacterized Function	C-light	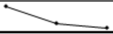	0.000	51.693	42.781	1.2
F1/F0 ATPase AtpABCDEFGH	D-light	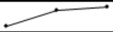	0.039	1.094	0.409	2.7
NAD(P)H:quinone oxidoreductase (complex I)	D-light	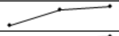	0.013	2.079	0.941	2.2
ribosome large subunit	D-light	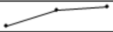	0.000	3.501	1.309	2.7
ribosome small subunit	D-light	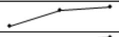	0.039	1.751	0.859	2.0
tRNA aminoacylation	D-light	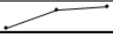	0.026	1.860	0.859	2.2
c-type cytochrome biogenesis_ system II	E-O_2	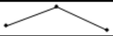	0.034	0.948	0.082	11.6
cytochrome b6-f complex	E-O_2	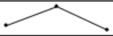	0.012	1.896	0.327	5.8
F1/F0 ATPase	E-O_2	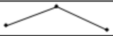	0.021	1.896	0.409	4.6
NAD(P)H:quinone oxidoreductase (complex I)	E-O_2	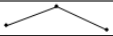	0.000	5.213	0.941	5.5
photosystem I main subunits	E-O_2	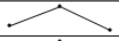	0.029	1.422	0.245	5.8
phycocyanin biosynthesis	E-O_2	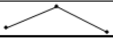	0.003	2.370	0.368	6.4
RNA polymerase sigma factors	F-O_2	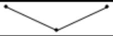	0.038	1.604	0.327	4.9
RND-type exporter Rnd3	F-O_2	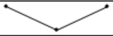	0.027	1.070	0.082	13.1
Sodium:hydrogen antiport	F-O_2	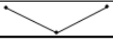	0.029	1.604	0.286	5.6
ribosome large subunit	G-O_2	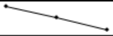	0.000	3.505	1.309	2.7
ribosome small subunit	G-O_2	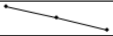	0.006	2.081	0.859	2.4
tRNA aminoacylation	G-O_2	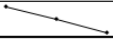	0.039	1.752	0.859	2.0
iron-sulfur cluster assembly SUF system	H-O_2	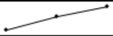	0.046	0.901	0.327	2.8
Uncharacterized Function	H-O_2	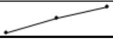	0.007	48.09	42.78	1.1
