# Supplementary material for: Indirect Interspecies Regulation: Transcriptional and Physiological Responses of a Cyanobacterium to Heterotrophic Partnership
Source: mSystems. 2017 Mar 7;2(2):e00181-16. doi: 10.1128/mSystems.00181-16 (PMC5340862; doi:10.1128/mSystems.00181-16)
Supplement: TABLE S2 [file sys002172092st10.pdf]

Table S2. Statistical/functional enrichment of *M. ruber* gene functions within clusters of mRNA profiles.

Subsystem	Cluster	Eigen-Gene	p-value	% in Module	% in Genome	Ratio
homoprotocatechuate degradation	A-light		0.033	1.158	0.209	5.5
peptides/nickel transport system PE	A-light		0.000	4.633	0.943	4.9
polar amino acid transport system PA	A-light		0.023	1.158	0.175	6.6
simple sugar transport system SS	A-light		0.044	1.158	0.244	4.7
translation elongation factor	A-light		0.015	1.158	0.140	8.3
xanthine degradation to urate	A-light		0.037	0.772	0.070	11.1
arsenical resistance system	B-light		0.021	1.064	0.070	15.2
omosome partitioning proteins_ inhibitors of FtsZ assen	B-light		0.048	1.064	0.140	7.6
iron homeostasis	B-light		0.021	1.064	0.070	15.2
lipopolysaccharide export system LptABCDEFG	B-light		0.015	1.596	0.209	7.6
transcription termination - antitermination Nus factors	B-light		0.033	1.064	0.105	10.2
adhesion	C-light		0.014	0.901	0.140	6.5
chromosome partitioning proteins	C-light		0.040	1.126	0.349	3.2
CRISPR immunity	C-light		0.025	2.252	0.943	2.4
iron (III) transport system AfuABC	C-light		0.035	0.901	0.209	4.3
NitT/TauT family transport system SN	C-light		0.015	1.126	0.244	4.6
phosphate signalling cascade PhoBRU-PstABCS	C-light		0.004	1.351	0.244	5.5
RND efflux pump	C-light		0.006	1.126	0.175	6.5
spermidine/putrescine uptake system PotABCD	C-light		0.025	1.351	0.419	3.2
TCA cycle second carbon oxidation	C-light		0.035	0.676	0.105	6.5
tetrahydrofolate biosynthesis from GTP and PABA	C-light		0.035	0.901	0.209	4.3
menaquinone biosynthesis via futalosine	D-light		0.027	1.235	0.279	4.4
phenylacetate degradation to succinyl-CoA	D-light		0.000	3.086	0.524	5.9
ribosome large subunit	D-light		0.000	4.012	1.152	3.5
ribosome small subunit	D-light		0.005	2.469	0.698	3.5
B12 biosynthesis late steps	E-O2		0.027	0.915	0.140	6.5
B12 salvage	E-O2		0.009	1.220	0.175	7.0
galactose degradation	E-O2		0.028	1.220	0.279	4.4
lactose uptake system LacEFGK	E-O2		0.017	0.915	0.105	8.7
peptidoglycan biosynthesis	E-O2		0.003	2.134	0.454	4.7
phenylacetate degradation to succinyl-CoA	E-O2		0.047	1.524	0.524	2.9
RND efflux pump	E-O2		0.009	1.220	0.175	7.0
spermidine/putrescine uptake system PotABCD	E-O2		0.000	2.439	0.419	5.8
lactate utilization	F-O_2		0.015	0.971	0.105	9.3
lipopolysaccharide export system LptABCDEFG	F-O_2		0.049	0.971	0.209	4.6
OMP chaperone system: SurA-Skp-DegP	F-O_2		0.050	0.647	0.070	9.3
simple sugar transport system	F-O_2		0.017	1.294	0.244	5.3
twin-arginine protein export system TatABCE	F-O_2		0.015	0.971	0.105	9.3
xanthine degradation to urate	F-O_2		0.050	0.647	0.070	9.3
zinc/manganese/iron(II) uptake system TroABCD	F-O_2		0.024	0.971	0.140	7.0
fructose uptake system FrcABC	G-O_2		0.040	0.641	0.105	6.1
NADH dehydrogenase I NuoABCDEFGHIUJKLMN	G-O_2		0.007	1.709	0.489	3.5
ribosome large subunit	G-O_2		0.005	2.991	1.152	2.6
TCA cycle	G-O_2		0.018	1.068	0.244	4.4
V-type ATPase AtpABCDEFGHIK	G-O_2		0.000	1.923	0.314	6.1
branched chain amino acid biosynthesis	H-O_2		0.015	1.034	0.175	5.9
peptides/nickel transport system PE	H-O_2		0.009	2.584	0.943	2.7
