# Supplementary material for: Indirect Interspecies Regulation: Transcriptional and Physiological Responses of a Cyanobacterium to Heterotrophic Partnership
Source: mSystems. 2017 Mar 7;2(2):e00181-16. doi: 10.1128/mSystems.00181-16 (PMC5340862; doi:10.1128/mSystems.00181-16)
Supplement: TEXT S1 [file sys002172092s3.docx]

**1. Cell counts and imaging.** Cell counting was performed by fluorescence activated cell sorting (FACS; see main text Materials and Methods) and cell counts are presented as the percentage of total counting events. Microscopic images were acquired on a Zeiss LSM 710 Scanning Confocal Laser Microscope (Carl Zeiss MicroImaging GmbH, Jena, Germany) equipped with a W Plan-Apochromatic 63x/1.0 M27 objective. *M. ruber* cells were visualized by SYBR Gold (Life Technologies, Carlsbad, CA) 499-598 nm. *T.* *elongatus* was visualized by auto-fluorescence measured at 651-721 nm. Images were processed with Volocity (Perkin Elmer, Waltham, MA) and used to obtain the cell size measurements made along the major and minor axis.

**2. Batch growth of *Meiothermus ruber***

Axenic *M. ruber* was cultured in batch to test for nitrate consumption. Cultures were grown in 250 ml shake flasks charged with 25 ml of BP1 media supplemented with 0.1% yeast extract, 5 mM lactate and 17 mM NaNO_3_. Cultures were inoculated with exponentially growing cells to an OD_600_ of 0.066, held at 55°C and agitated at 150 RPM for 48 hrs. An abiotic control was run under the same conditions. Nitrate was measured on a Dionex ICS-200 anion chromatograph (Thermo Fisher Scientific Inc., Waltham, MA ) ([1](#_ENREF_1)).

Axenic *M. ruber* was unable to consume nitrate (Fig. S2), which was the sole nitrogen source supplied during cultivation of the binary consortium. This experimental result is complemented by the fact that the *M. ruber* genome does not contain the principal genes associated with nitrate reduction (assimilatory or dissimilatory). *M. ruber* was reliant upon organic nitrogen supplied by *T. elongatus* during binary cultivation.

**3. Metabolite Analysis**

Samples were collected from the turbidostat during steady-states (biological duplicates sampled 1 or more residence times apart from each other) and passed through a 0.2 µm syringe filter to collect a spent media fraction. Then, 1 mL filtrate aliquots were was dried under a speed-vac concentrator for chemical analysis. Fresh sterile medium was prepared by the same method for control. Dried samples were chemically derivatized via methoxyamination and trimethylsilyation, and analyzed by GC-MS as reported previously ([2](#_ENREF_2)). Metabolite peaks which were detected over the fresh medium samples were monitored and plotted with their intensities. The assay screened for sugars (e.g., sucrose), organic acids and amino acids (Fig. S3). Extracellular metabolite signatures for sugars were not distinguishable from the fresh/sterile media controls and citric acid was the only organic acid identified to change. Interestingly, a suite of amino acids were detected between the axenic *T. elongatus* and the binary culture conditions.

**4. RNA isolation and sequencing for axenic *T. elongatus***

Axenic *T. elongatus* experiments were initially analyzed and compared prior to performing binary cultivation experiments with *M. ruber*. Binary culture samples were composed of approximately <10% *M. ruber* by cell count. Therefore, to increase the quality of *M. ruber* transcriptomic data the extraction and sequencing methodology used was modified. Sample prepared by the following methods were only used to examine *T. elongatus* genes and their changes in expression during axenic versus binary growth.

Samples were collected from the turbidostat during steady-states. RNA was extracted using Invitrogen TRIzol® Reagent (Thermofischer, Waltham, MA), followed by genomic DNA removal and cleaning using Qiagen RNase-Free DNase Set kit (Qiagen, Hilden, Germany) and Qiagen Mini RNeasy™ kit (cat#74104). The Agilent 2100 Bioanalyzer was used to assess the integrity of the RNA samples. Only RNA samples having RNA Integrity Number (RIN) between 8 -10 were used. The Applied Biosystems SOLiD^TM^ Total RNA-Seq kit (catalog number 4445374) was used to generate the cDNA template library according to the instruction manual. The SOLiD^TM^ EZ Bead system was used to perform emulsion clonal bead amplification to generate bead templates for SOLiD^TM^ platform sequencing. Samples were sequenced on the 5500XL SOLiD^TM^ platform. The 50-base short read sequences produced by the 5500XL SOLiD^TM^ sequencer were mapped in color space using SOLiD^TM^ LifeScope^TM^ software version 2.5 using the default parameters against genome of *Thermosynechococcus elongatus* BP-1 DNA NC-004113, both the fasta and the GFF files can be obtained from NCBI Genome databases (<http://www.ncbi.nlm.nih.gov/genome>). Raw counts from SOLiD sequencing were then normalized with DESeq2 ([3](#_ENREF_3)) and differentially expressed genes (defined as >2.0 fold change with an adjusted *p*-value of < 0.05) were also identified with this same program.

**5. Transcriptomic analyses**

Messenger RNA abundance profiles, given in RPKM values for each steady-state condition (repeated as biological duplicates), were filtered to remove any gene with an average count of zero in any condition and any gene with an RPKM value of < 15 when all conditions were averaged. Binary culture expression profiles were identified via K-means clustering and calculated with the custom Matlab script (below) which masked the bottom 30% of genes with the smallest variance across each respective profile (i.e., flat profiles). Significant enrichment was defined as the percentage of genes within the profile for which a function has been assigned being significantly higher than the percentage of genes of the same function in the entire genome with a *p*-value of < 0.05 according to Fisher’s exact test.

**6. Growth and photosynthesis kinetics**

The net rate of O_2_ production was calculated from the steady-state mass balance through the bioreactor control volume (Equation S1).

$q_{O_{2}}x= D\left( {[O}_{2}^{in\_aq}]-[O_{2}] \right)+k_{l}a\left( k_{H}pO_{2}-[O_{2}] \right)$ Eq. S1

The specific rate of O_2_ production (q_O2_) multiplied by the biomass concentration (x) is interpreted here as the net rate of O_2_ production during photosynthesis ([4](#_ENREF_4)) and is a function of the dilution rate (D), k_l_a (17.6 ± 0.2 h^–1^,), dissolved O_2_ concentration ([O_2_]), partial pressure of O_2_ within the sparge gas (pO_2_) and Henry’s law partitioning coefficient (k_H_ = 8.07 ∙ 10^–4^ mol L^–1^ ATM^–1^; at 52°C). The specific rate of biomass production (q_x_; Cmmol biomass h^–1^·g^-1^_AFDW_) was calculated by assuming the molecular weight of dry biomass to be 24.59 g_AFDW_·Cmol^-1^ ([5](#_ENREF_5)) .

**7. Statistical tests**

Statistical tests were used to interpret the data presented in the main text. The slopes presented in Figure 1C and Figure 2 were determined to be significantly different from each other with > 99% certainty. This was determined using a t-test for unequal sample sizes assuming equal variances, Equations S2 and S3; where *Y_i_*, *n_i_*, and *S_i,j_* are the respective slopes, number of averaged data points and standard error, respectively.

$t_{statistic}= \frac{Y_{1,avg}-Y_{2,avg}}{S_{1,2}\sqrt{\frac{1}{n_{1}}-\frac{1}{n_{2}}}}$ Eq. S2

$S_{1,2}= \sqrt{\frac{\left( n_{1}-1 \right)\cdot s_{1}^{2}+\left( n_{2}-1 \right)\cdot s_{2}^{2}}{\left( n_{1}-1 \right)+\left( n_{2}-1 \right)}}$ Eq. S3

A Tukey’s range test ([6](#_ENREF_6)) was used to interpret the difference between average values presented in Figure 3 which were also determined to be significantly different from each other with > 99% certainty.

**8. MATLAB K-means clustering script.**  Data inputs to reproduce this clustering analysis can be derived by importing the Excel files provided as supplementary data to the manuscripts. Some parsing is needed to satisfy the input structures specified as comments in the code under %input.

function [Cdata,colhead,clusts]=BP1Kmeans(rpkm,genes,cond)

%Written by Hans C. Bernstein 2/10/15

%Matlab 2014

%hans.bernstein@pnnl.gov

%Citation: Mathworks "Gene Expression Profile Analysis"

%www.mathworks.com/help/bioinfo/examples/

%REQUIRES MATLAB STATISTICS/BIOINFORMATICS TOOLBOX

% performs Kmeans clustering on normalized mRNA abundances (e.g.rpkm)

%input

% rpkm [matix m x n] containing gene expression in rpkm or other

% genes <n x 1 cell> of strings identifying the rows (row)

% cond <1 x m cell> of strings identifying the profile conditions (column)

%output

%Cdata <m x (n+2) dataset> containing all genes, cluster IDs & rpkm values

%colhead <1 x (n+2) cell> contains column headers for Cdata

%clusts [matrix 4 x n] contains profile of each cluster's centroid

%%%%%%%%%%%%%%%%%%%%%%%%%%%%%%%%%%%%%%%%%%%%%%%%%%%%%%%%%%%%%%%%%%%%%%%%%%%

g=genes; %store var "g" to keep original, unfiltered gene list

fprintf('number of original genes:');

length(genes)

%% pre processing

%remove "empty spots"

emptySpots=strcmp('EMPTY',genes);

rpkm(emptySpots,:)=[];

genes(emptySpots)=[];

%% MORE pre processing

%remove genes w/ "NaN" values

nanIndicies = any(isnan(rpkm),2); % isnan identifies genes w/ missing data

rpkm(nanIndicies,:)=[];

genes(nanIndicies)=[];

%% MORE pre processing

%replace zero expression values

R=zeros(length(rpkm(:,1)),length(rpkm(1,:)));

m=1;

while m <= length(rpkm(:,1));

n=1;

while n <= length(rpkm(1,:));

if rpkm(m,n) ~= 0;

R(m,n)=rpkm(m,n);

end

if rpkm(m,n)==0;

R(m,n)=0.5; %sets value below possible (non-zero) min RPKM

end

n=n+1;

end

m=m+1;

end

%% more pre processing OPTIONAL!!!

%filter "flat profiles"

%filters out genes w/ small variance over the profile

mask=genevarfilter(R,'Percentile',30);

% Use the mask as an index into the values to remove the filtered genes

R=R(mask,:);

genes=genes(mask);

%% more pre processing

%Normalize the rpkm values by the mean of each gene profile

N=zeros(length(R(:,1)),length(R(1,:)));

m=1;

while m <= length(R(:,1));

av=mean(R(m,:));

n=1;

while n <= length(R(1,:));

N(m,n)=R(m,n)/av;

n=n+1;

end

m=m+1;

end

fprintf('number of unfilterd genes:');

length(genes)

%% Log base 2 transformation

L2val=log2(N); %calc log base 2 of RPKM values

%% perform K-means clustering

rng('default'); %initialize random number generator

[cidx, ctrs]=kmeans(L2val,4,'dist','corr','rep',5,'disp','final');

%% Plot clusters

%generate arbitrary abscissa vector w/ sequentially increasing numbers

i=1;

while i <= length(cond);

ab(i)=i;

i=i+1;

end

figure;

for c = 1:4;

subplot(1,4,c); %THIS CAN BE MODIFIED TO OUTPUT CUSTOM SUBPLOT ARRAY

plot(ab,L2val((cidx == c),:)); % PLOTS ALL PROFILES

hold all;

% PLOTS PROFILE OF EACH CLUSTER'S CENTROID

plot(ab,ctrs(c,:),'-k', 'lineWidth', 5);

axis tight;

axis('square');

set(gca,'XTick',[1 2 3],'XTickLabel',cond,'fontname','symbol');

end

suptitle('K-means Clustering of Profiles');

%% create cluster data object

clustID=mat2dataset(cidx);

totgeneID=mat2dataset(g);

geneID=mat2dataset(genes);

RPKM=mat2dataset(rpkm);

z=mat2dataset(zeros(length(g),1)); %create ID "0" for genes not clustered

%concatenate all genes, cluster IDs and RPKM vals

totdata=[totgeneID z RPKM];

data=[geneID clustID];

%combine filtered and unfiltered data

i=1;

while i <= length(g)

j=1;

while j <= length(genes)

t=isempty(strfind(g{i},genes{j}));

if t == 0;

totdata(i,2)=data(j,2);

end

j=j+1;

end

i=i+1;

end

colhead={'genes' 'clusterID'};

colhead=[colhead cond]; %create cell array for column headers

Cdata=totdata; %full data set w/ cluster IDs

clusts=ctrs;

fprintf('Kclust_prof ran in (seconds):');

cputime

%eof

**9. Supplementary References Cited**

1. **Pfaff J, Brockhoff C, O’Dell J.** 1991. The determination of inorganic anions in water by ion chromatography. US Environmental Protection Agency Method **300**.

2. **Kim Y-M, Nowack S, Olsen MT, Becraft ED, Wood JM, Thiel V, Klapper I, Kühl M, Fredrickson JK, Bryant DA.** 2015. Diel metabolomics analysis of a hot spring chlorophototrophic microbial mat leads to new hypotheses of community member metabolisms. Frontiers in microbiology **6**.

3. **Love MI, Huber W, Anders S.** 2014. Moderated estimation of fold change and dispersion for RNA-seq data with DESeq2. Genome Biol **15:**1-21.

4. **Bernstein HC, Kesaano M, Moll K, Smith T, Gerlach R, Carlson RP, Miller CD, Peyton BM, Cooksey KE, Gardner RD.** 2014. Direct measurement and characterization of active photosynthesis zones inside wastewater remediating and biofuel producing microalgal biofilms. Bioresource technology **156:**206-215.

5. **Roels J.** 1980. Application of macroscopic principles to microbial metabolism. Biotechnol Bioeng **22:**2457-2514.

6. **Tukey JW.** 1949. Comparing individual means in the analysis of variance. Biometrics**:**99-114.
